# Navigating the cancer care continuum: A comparative study of Black and White breast cancer patients

**DOI:** 10.1371/journal.pone.0312547

**Published:** 2024-10-24

**Authors:** Min K. Lee, Naomi T-T. Levine, Lisa R. Hayes, Cleveland G. Shields, Yuehwern Yih

**Affiliations:** 1 School of Industrial Engineering, Purdue University, West Lafayette, IN, United States of America; 2 Independent Scholar, Bremerton, WA, United States of America; 3 Pink-4-Ever Ending Disparities, Indianapolis, IN, United States of America; 4 Department of Human Development and Family Studies, Purdue University, West Lafayette, IN, United States of America; 5 LASER PULSE (Long-Term Assistance and SErvices for Research, Partners for University-Led Solutions Engine) Consortium, Purdue University, West Lafayette, IN, United States of America; Lamar University, UNITED STATES OF AMERICA

## Abstract

Despite improvements in early detection and therapeutic interventions, the mortality rate for Black breast cancer patients is still significantly higher than that of White breast cancer patients. This study seeks to understand differences in the patient experience that lead to this disparity. Semi-structured interviews were conducted to understand the breast cancer treatment process and patient experiences. This study collected health services and timeline data from medical records. Based on these two data sources, the patient’s journey in breast cancer treatment was mapped and a thematic analysis was conducted to identify challenges and barriers in the process. The cancer care continuum consists of four stages—diagnosis, surgery, chemotherapy/radiation, and follow-up care. The themes contributing to patient experiences and challenges were identified and compared in each stage for both Black and White patients. Both Black and White participants faced challenges related to financial constraints, treatment changes, lack of autonomy, and insufficient emotional support. However, Black participants additionally faced significant barriers in terms of cultural concordance, effective patient-provider communication, and delay in diagnosis. This study highlights the importance of incorporating effective provider-patient communication, navigation, and emotional support, especially for Black breast cancer patients throughout the cancer care continuum to address healthcare disparities.

## Introduction

Breast cancer is the most commonly diagnosed form of cancer and the second leading cause of death among women in the United States [1]. Each year, over 280,000 new cases are reported, making up nearly 30% of all new cancer cases in the country, accompanied by more than 43,000 deaths [2]. Nonetheless, extensive research over the decades has illuminated the intricate nature of breast cancer and has contributed to enhanced clinical results [3–5]. Progress in early detection and therapeutic interventions has led to a decline in breast cancer mortality in the U.S. and various developed countries over the past few decades [6].

Unfortunately, this decrease in mortality is not uniform across populations. In the U.S., the age-adjusted breast cancer mortality rates are roughly 40% higher in Black women compared to non-Hispanic White women, even though Black women have a lower incidence rate [3]. Black women often face more advanced and aggressive forms of the disease, such as the triple-negative phenotype, and have a lower 5-year survival rate at every disease stage [4]. Additionally, they are more frequently diagnosed with comorbid conditions like hypertension, diabetes, and heart disease compared to their White counterparts [5].

The literature on racial disparities in breast cancer outcomes highlights significant differences in the patient experience, particularly regarding patient voice and patient-centered care. Black patients often encounter shorter and less detailed conversations with their clinicians compared to their White counterparts, which can hinder effective communication and shared decision-making [7]. Research indicates that Black patients often receive less information about their treatment options and prognosis, resulting in reduced participation in their own care and diminished empowerment to make informed healthcare decisions [8–10]. Furthermore, those who receive clear guidance are more likely to adhere to appropriate follow-up actions after an abnormal mammogram [11]; however, inadequate communication is disproportionately experienced by Black women compared to White women [12].

This disparity in communication is further compounded by racial discordance between patients and providers, where Black patients are more likely to report dissatisfaction with the care they receive from racially discordant clinicians [8]. The underrepresentation of Black nurses and clinicians in the healthcare system may contribute to structural racism, leading to challenges in establishing trust and rapport with Black patients [7].

The concept of ‘patient voice’ encapsulates the multi-dimensional challenges patients face–physical, emotional, and cognitive–underscoring the need for patient-centered care that fully considers the entire treatment journey [13, 14]. While there is a growing body of literature addressing racial disparities in specific aspects of the breast cancer journey, a significant gap remains in understanding the experiences that span the entire treatment process [15]. Comprehensive studies that map out the entire experience are instrumental in ensuring personalized, empathetic, and effective care across all stages of the disease.

This study seeks to bridge existing knowledge gaps by comparing the experiences of Black and White breast cancer patients throughout their journey. By investigating the breast cancer patient’s journey and incorporating their personal experiences and expectations at each stage of the disease, our research not only offers insights that can reshape healthcare policies but also enhances patient care and outcomes. Furthermore, the findings can foster better relationships between the medical system, care teams, and patients, ensuring resources are tailored to patients’ needs, ultimately improving their quality of life. This study focuses on Indianapolis, a metropolitan area in the state of Indiana with a 29% Black population [16]. Consistent with the literature, despite improvements in the screening process, the gap in mortality rate between Black and White breast cancer patients has been persistent in Indiana [17, 18].

## Materials and methods

### Study design and data collection

In this qualitative research, we examined the conventional care pathway for women battling breast cancer to develop a comprehensive schematic that visually encapsulates their journey. An interview guide was created based on breast cancer literature and was adapted in collaboration with the Reaching to End Disparities (R.E.D.) Alliance (now known as Pink-4-Ever Ending Disparities). R.E.D. Alliance is a non-profit organization founded with the mission to empower, educate, and support breast cancer survivors, caregivers, and families, addressing breast health disparities and providing a range of services to aid individuals affected by breast cancer. A detailed outline of the interview questions is provided in S1 File. Demographic and health information was collected at the time of interview scheduling. We have also obtained protected health information (PHI) from the survivors’ health service providers to verify their progression timeline through the cancer care continuum. These medical records were accessed until November 2023 for research purposes.

### Recruitment

Inclusion criteria included being female, over 18 years of age, a past diagnosis of breast cancer, and good mental status. Participants were recruited through the R.E.D. Alliance’s established collaborations with researchers, medical professionals, and cancer advocacy organizations and relationships with breast cancer survivors and women in the faith-based community. A study description was sent to potential participants through the R.E.D. Alliance mailing list and its social media platforms. Those expressing interest provided permission for their contact information to be shared with the principal investigator, who conducted all interviews. The investigator then reached out to those women, offering a more in-depth explanation of the study, and addressing any questions. Upon agreement to participate, an appointment was scheduled for either a videoconference or an in-person visit.

A total of 11 women voluntarily participated in this research. Interviews took place between February 2020 and January 2022, each lasting approximately 60 to 90 minutes. All interviews were audio-recorded and subsequently transcribed verbatim. Demographic and health information was gathered when scheduling the interviews, with variables including demographics and family history of cancer (Table 1).

**Table 1:** Sociodemographic and clinical characteristics: A comparison between Black and White breast cancer study participants.

		Black (n = 6)	White (n = 5)
Current age in years, median (IQR)		60 (54.5, 62.5)	58.5 (43.3, 71)
Employment Status, n (%)	Employed full-time	4 (67%)	3 (60%)
	Not Employed	2 (33%)	2 (40%)
Marital Status, n (%)	Not married/Divorced	1 (17%)	2 (40%)
	Married	5 (83%)	3 (60%)
Education, n (%)	High school	0 (0%)	1 (20%)
	4-year college degree	5 (83%)	2 (40%)
	Post-graduate degree	1 (17%)	2 (40%)
Family History, n (%)	Yes	5 (83%)	5 (100%)
	No	1 (17%)	0 (0%)
Household Income, n (%)	30,000–59,999	0 (0%)	2 (40%)
	60,000–89,999	3 (50%)	1 (20%)
	90,000–149,999	3 (50%)	1 (20%)

IQR, Interquartile Range; n, number of participants; the percentages do not add up to 100% because some participants chose not to answer.

This study received approval from the Purdue Institutional Review Board (IRB #1811021261). Each participant signed an informed consent form, authorizing her participation and permitting the limited release of portions of her interview in a way that ensured confidentiality. The findings in this report have been presented while upholding the confidentiality of all study participants.

### Data analysis

Interview data were analyzed using qualitative thematic analysis. Analyses were conducted following the framework of Ciria-Suarez et al. and Mitchell et al. [19, 20]. Interview transcripts were thoroughly reviewed to discern recurring themes from the women’s experiences with cancer. Two researchers analyzed the transcripts independently, each identifying key themes, patterns, and concepts. They then collaborated to develop a unified codebook. Codes were grouped by similarities and differences, forming overarching themes. To ensure robustness, the team compared these insights with fixed data like sociodemographic and clinical characteristics. For each theme, representative quotes were determined separately for Black and White participants. The team reached a unanimous agreement on the theme categorization, and representative quotes were selected separately for Black and White participants to highlight these themes.

### Researcher positionality

There are five authors for the current study. The primary author, who identifies as an ethnic minority, led the data collection and analysis, utilizing background in participatory action research and experience working with communities to address disparities in healthcare access. The second author, who identifies as White, has extensive experience in addressing disparities in healthcare access, contributed to the thematic analysis. The third author, an ethnic minority and breast cancer survivor, brought significant experience in breast cancer advocacy, particularly within marginalized communities, which was essential for contextualizing the findings. The fourth author, who identifies as White, offered valuable insights with his expertise in disparity of healthcare communication and patient-centered care. The fifth author, an ethnic minority scholar with expertise in systems engineering, leading this research project, contributed to the identification of the research question, overall study design and analysis, and offered perspectives on the broader structural issues affecting healthcare outcomes. All authors contributed to interpreting the findings and their implications. We acknowledge, however, that our backgrounds may have influenced our interpretations of the data. To mitigate this, we made deliberate efforts to bracket any existing biases or assumptions, documenting preconceptions during the data collection and analysis process to ensure our findings remained true to the participants’ voices.

## Results

### Participants

In total, 11 women with a median age of 60 (Interquartile Range (IQR): 49.25–65.25) were interviewed. Most of the study population was married (73%), had a college education (64%), had a family history of cancer (91%) and were employed full-time (64%). Table 1 shows a comparison of sociodemographic and clinical characteristics between Black and White breast cancer study participants.

### Breast cancer patient journey map

The journey for women diagnosed with breast cancer is intricately shaped by distinct medical stages, commonly framed within the cancer care continuum. This continuum represents the sequence of steps that people navigate from diagnosis through follow-up care. Each phase comes with its own set of unique experiences, eliciting specific physical, emotional, cognitive, and social responses. Many patients perceive this trajectory as a predefined path–a necessary course to potentially save their lives–that is largely dictated by the type and stage of their cancer. However, individuals may enter the cancer care continuum at any stage and aim to continue through it without delay. Table 2 outlines the differences in themes and subthemes experienced by Black and White participants throughout various stages of the breast cancer journey, as identified in this study.

**Table 2:** Thematic differences in the breast cancer journey between Black and White participants at each stage.

Stage	Themes	Subthemes	Black	White
Throughout	Emotional Response	Absence of emotional support from the treatment team	X	X
	Support network	Support from loved ones	X	X
		Employer support	X	X
Diagnosis	Delayed detection and diagnostic barriers	Diagnostic failures	X	
		Delays due to lack of communication and follow-up	X	
		Insurance and financial barriers to diagnosis	X	X
	Communication and Information Management	Information overload	X	X
		Lack of effective communication	X	X
	Shared experience and cultural connection	Need for cultural competency among healthcare providers	X	
		Need for connections with support networks	X	
Surgery	Insurance and Financial barriers	Financial constraints in treatment access	X	
		Challenges in insurance navigation		X
	Unmet expectations and treatment changes		X	X
	Patient autonomy and involvement in care decisions	Lack of sufficient information for informed decision making	X	X
		Lack of control over scheduling and treatment plans	X	X
	Lack of communication and accessibility of providers		X	X
Chemotherapy/Radiation	Managing treatment side effects	Physical discomfort and fatigue	X	X
		Lack of preparedness for side effects	X	
	Balancing illness with daily life	Juggling multiple roles	X	X
		Role of caregiving and family responsibilities	X	
Post-treatment	Treatment fatigue and decision to discontinue	Emotional and physical exhaustion	X	
		Dissatisfaction with healthcare providers	X	
	Patient autonomy	Increased empowerment	X	X
		Confidence in decision making	X	X
	Lack of follow-up care and continued support		X	X

‘X’ indicates that participants in the respective group reported experiencing or identifying the theme or subtheme during their breast cancer journey.

**Fig 1:**
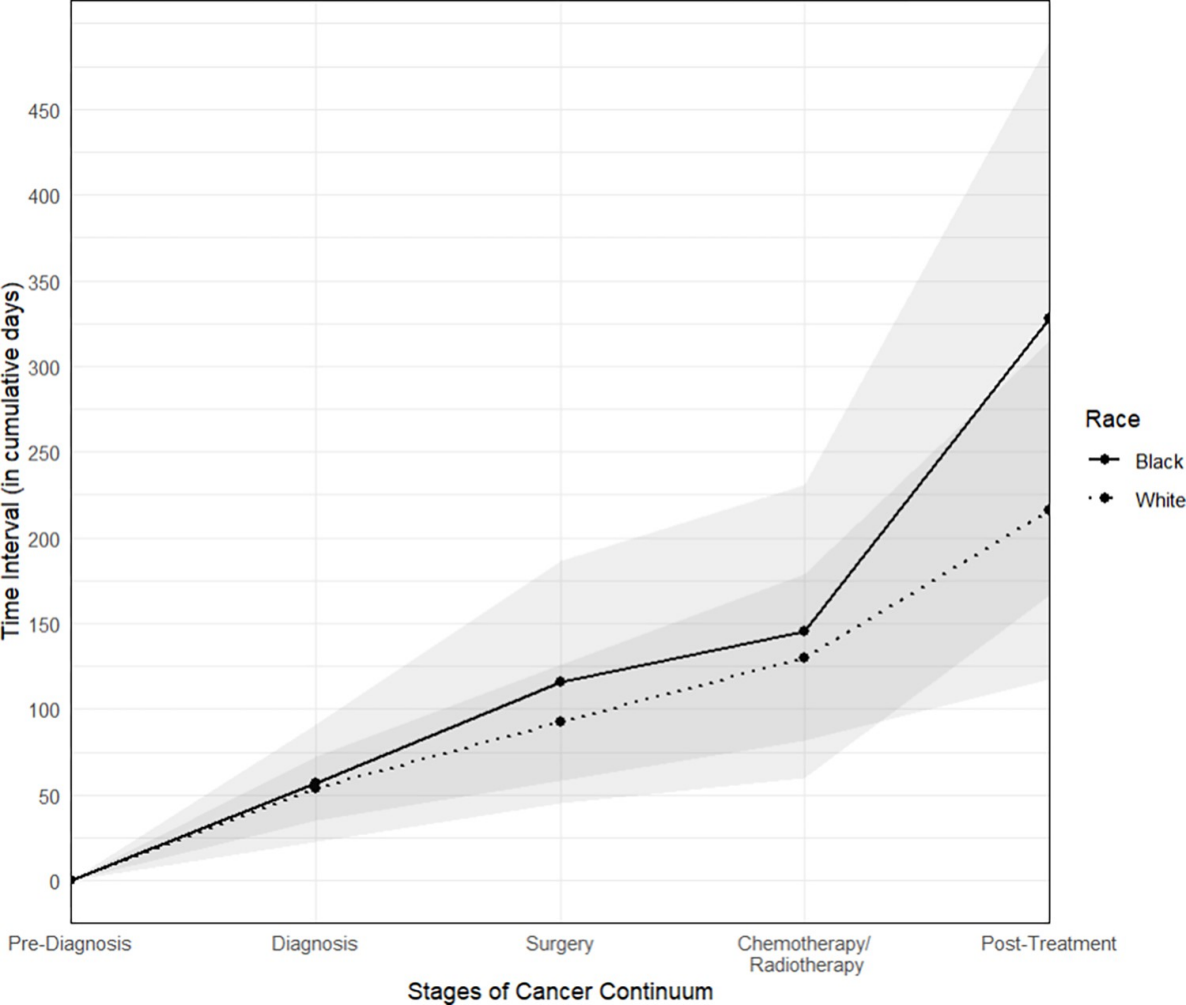
Time intervals in cancer care continuum among study participants. The gray shades indicate the confidence intervals for the time intervals between each stage of the cancer care continuum.

Fig 1 presents a plot illustrating the average time intervals between different stages of the cancer care continuum. The time delays are greater for Black participants at many of these stages and exhibit higher variability.

#### Throughout the cancer care continuum

The cancer care continuum represents the entire trajectory of cancer care, from the moment of diagnosis through the end of treatment and beyond into survivorship. There were two themes in the interview responses that reflected the overall care continuum: emotional response and support network.

*Emotional response*. Throughout the continuum, the emotional response becomes a critical aspect of the patient’s experience. Emotional support—or the lack thereof—is a theme that resonates across all patient experiences. Both Black and White participants reported feelings of isolation and a deficit of compassionate care within the healthcare system.

“*I received zero emotional support from the treatment team from screening to treatment. I didn’t know what to expect…I didn’t know the professional supports were available at that time.”* (Participant 1; Black participant)

“*In the beginning*, *you are overwhelmed*. *There is a different swirling of emotions*, *and that’s not really a comfortable thing to have to bring up*. *I feel like I needed more emotional support in the beginning*. *I didn’t get that towards halfway in*.” (Participant 7; White participant)

“*Within the hospital*, *there was no one I could turn to*, *to help with my fear*. *I don’t think any of the treatment team was addressing the emotional side*” (Participant 10; Black participant)

*Support network*. To cope with these challenges, family and friends emerged as one of the elements most intricately involved in the disease process. Both Black and White participants disclosed a broad range of support they received from family and friends, illustrating the crucial role loved ones play in providing emotional sustenance and practical assistance through the treatment journey.

“*When I was sick, my daughter was the caregiver. She made sure everything I needed was next to me*.” (Participant 1; Black participant)

“*The first time you go through*, *you’ll be so frightened and there will be lots of things that you will forget*. *You really need someone to help you go through this together*.” (Participant 6; White participant)

Many participants also revealed a significant level of gratitude towards understanding and accommodating employers during their health crisis.

“*My boss, who was female and whose mother had a cancer issue, was informed. My boss was really helpful and asked me if I would like to be in FMLA or continue working. After 18 months of sick leave, I came back to work. All in all, my boss was supportive*.” (Participant 1; Black participant)

“*I had FMLA during my chemotherapy*, *which made my financial stable*. *It had no impact on my income and insurance*.*”* (Participant 5; White participant)

#### Diagnosis

The diagnosis stage involves a battery of medical tests and screenings that enabled the identification of the disease. This stage is foundational in forming the treatment strategy based on the cancer type and stage. There were three themes in the interview responses that reflected this stage in the care continuum: delayed detection and diagnostic barriers, communication and information management, and shared experience and cultural connection.

*Delayed detection and diagnostic barriers*. During the diagnosis stage, patients encounter obstacles in accessing timely healthcare services and information. This barrier necessitates a degree of self-advocacy, which manifests distinctly across demographics. For Black participants, this self-advocacy was particularly pronounced, and it often arose from perceived neglect or oversight by their healthcare providers.

“*Despite routine screening*, *cancer was not detected*. *I discovered it myself when I felt some dimpling in my right breast*.” (Participant 4; Black participant)

“*Although previous mammo showed dense tissues*, *no one had talked to me about possible indications or possible treatment options*.*”* (Participant 8; Black participant)

“*I felt a lump and scheduled appointment for regular mammogram*, *not diagnostic mammogram*, *which needs doctor’s approval*. *The doctor approved it on the same day*, *but I still had to wait a week*. *I don’t know why they sent me back when they could just call the doctor*. *The waiting time was like eternity*. *My mom and sister had it*, *so I was pretty sure that it was cancerous*.*”* (Participant 11; Black participant)

In contrast, White participants also demonstrated self-advocacy, but this was more focused on navigating systemic barriers related to insurance and finding alternative pathways to care, themes that both White and Black participants faced.

“*Insurance didn’t cover 3D mammogram, so I went with regular screening. It did cover genetic testing, so I did do this.*” (Participant 2; White participant)

“*My physician sent the paperwork for additional mammogram but the nurse told me that it will not be covered by insurance as I have already had mammogram a few months ago*.” (Participant 4; Black participant)

“*In late 2020*, *another lump was discovered*, *and I was recommended to get an MRI*. *My insurance wouldn’t cover MRI*, *so I had to price-shop to find an affordable MRI*. *I had my MRI done in early 2021*.” (Participant 9; White participant)

*Communication and information management*. As patients navigate these complexities, communication and information management become critical. The efficiency and clarity of the exchange between healthcare providers and patients are critical, yet both Black and White participants reported feeling overwhelmed by the deluge of information.

“*I was given a binder, but it was too much information at the beginning. I was not sure what estrogen/HER2 negative or positive meant. There was just so much in the air.*” (Participant 4; Black participant)

“*I felt like they tried to combine some of my appointments if they could*. *And because of that I was overwhelmed with information*. *I was extremely overwhelmed*.” (Participant 7; White participant)

“*Throughout the process*, *there is a lot of information coming at you at one time*. *Sometimes I don’t know what questions to ask*.” (Participant 8; Black participant)

*Shared experience and cultural connection*. This theme underscores the patients’ desire for healthcare professionals to resonate with their cultural background and personal experiences. The significance of this theme is particularly acute for Black participants, who consistently underscore the importance of representation and cultural competency in healthcare settings.

“*It would be nice to connect with other breast cancer patients on a more personal level, right from the time of diagnosis. It would have been helpful if someone on the treatment team, like doctors or nurses, had initiated the conversation about talking to different groups of people.*” (Participant 1; Black participant)

“*I believe the African American woman (nurse practitioner) made the difference because of the comfort level and shared experience*. *I spent most of my time discussing with her throughout my care*.” (Participant 4; Black participant)

“*I wanted a Black doctor because I felt that they would understand my skin better*. *I wanted to be able to have that dialogue with ease and be comfortable*.” (Participant 10; Black participant)

#### Surgery

In this stage, patients undergo surgical procedures aimed at removing the tumor and potentially some surrounding tissue. Depending on the extent and type of cancer, different surgical methods may be employed. There were four themes in the interview responses that reflected this stage in the care continuum: insurance and financial barriers, unmet expectations and treatment changes, patient autonomy and involvement in care decisions, and communication and accessibility of providers.

*Insurance and financial barriers*. Both Black and White participants expressed concerns and challenges related to insurance and finances. Navigating these barriers is often intertwined with systemic inequalities, leading to discrepancies in treatment availability or affordability based on the type of insurance they had.

“*People were getting different answers on treatment plan depending on what insurance they have. It was based on my ability to pay.*” (Participant 4; Black participant)

“*Whether or not to do the BRCA testing was based all on the cost of it*. *I heard from someone that it costed $10*,*000 so [he/she] didn’t do it*. *So that kind of number was floating around my head when they started talking about how much it was going to cost if I don’t meet the criteria*.” (Participant 8; Black participant)

“*The biggest barrier was from a financial perspective because of the hospital and their coding…There was a lot of back and forth to set it up with my insurance company*.” (Participant 9; White participant)

*Unmet expectations and treatment changes*. The treatment process can deviate from initial plans. This divergence can introduce additional uncertainty into an already complex treatment landscape, necessitating adaptability, and resilience from patients and providers alike.

“*I feel like I was given false hopes. None of the plans happened as expected.*” (Participant 7; White participant)

“*I went in (surgery) thinking I was at stage 1 or stage 2*, *but it was actually stage 3*. *They told me I had two small spots*, *but it was actually 10*.*4 cm*. *They discovered it only during the surgery*. *I had to have a second surgery*. *It was very painful*.” (Participant 4; Black participant)

*Patient autonomy and involvement in care decisions*. Despite the often-unpredictable nature of the treatment process, a recurring concern among patients is the lack of involvement in the decision-making process. Both Black and White participants have expressed that they were not sufficiently informed or integrated into the decision-making process, and that they had little control over their appointments.

“*I wish I would have gotten more information and learned more about the treatment options. You know that would have played more into my discussion of when and where they would start.”* (Participant 7; White participant)

“*The nurse navigator was talking about the surgeon’s schedule*, *and it seemed like the surgeon wanted to get it done before his vacation*. *I got a feeling that he wants to rush before he goes on vacation and relax thereafter*.*”* (Participant 8; Black participant)

“*We (the surgeon and I) were just talking about treatment options and then he did the MRI*. *I don’t know if he saw anything new*, *but he just said*, *you know*, *I’ve got to schedule you for this*. *So*, *it seemed like I was not getting enough information*.*”* (Participant 8; Black participant)

*Communication and accessibility of providers*. The theme highlights the struggle patients face in establishing and maintaining open lines of communication with their healthcare providers. Both Black and White participants showed frustration related to unanswered calls and unreturned voicemails, leading to emotional distress during a vulnerable time.

“*The doctor was not available when I had questions; [his/her] manner was very bad, was not supportive, and was not available most of the time.”* (Participant 4; Black participant)

“*The nurse navigator would be the obvious person to call but I couldn’t call her directly*. *I had to leave a voicemail and she didn’t return my calls half of the time*.” (Participant 5; White participant)

“*A week before my surgery*, *I called the nurse*, *and she was off for the week*. *Nobody called me and I was so scared…I spoke to probably two or three different nurses*. *But there was no follow-up even after I had the surgery*. *It was horrible*. *It would have been nice for her to think about the journey that I’m on*.*”* (Participant 10; Black participant)

#### Chemotherapy/radiation

This stage involves intense treatments that can have strong side effects. Patients often encounter physical and emotional challenges that affect quality of life. There were two themes in the interview responses that reflected this stage in the care continuum: managing treatment side effects and balancing illness with daily life.

*Managing treatment side effects*. The patient’s experiences highlight the physical toll of the treatments and the importance of managing these side effects to maintain some quality of life. Many participants indicated that they have suffered physical discomfort such as fatigue, pain, and nausea.

“*After chemotherapy, I was really sick and couldn’t get up from bed. I was angry, but I understood that it was what I needed to do, and I didn’t want to be a complainer.*” (Participant 1; Black participant)

“*I had a number of different side effects*… *I didn’t know where it was coming from*, *but it was coming very slowly*. *But eventually*, *I said*, *No I can’t*, *I can’t do that*.” (Participant 4; Black participant)

“*I had skin infection from chemo*. *Somehow my immune system was down*. *It was horrible*.” (Participant 7; White participant)

However, they encountered a gap in understanding the potential adverse impacts of treatments or how to cope with the adverse impacts.

“*I do not feel that I was given as much information that I needed to help myself prepare for this.*” (Participant 4; Black participant)

*Balancing illness with daily life*. Both Black and White participants illustrated the diverse approaches to balancing the demands of treatment with personal and professional life, and how individual circumstances can significantly shape this experience.

“*During chemo, my daughter worked at night, so I took care of my granddaughter. I felt like having the responsibility to take care of my granddaughter was really helpful. She kept me more focused. The responsibility made a difference.*” (Participant 1; Black participant)

“*While I was doing chemo*, *I was still a full-time mom*, *and I was still juggling being a full-time employee*.” (Participant 7; White participant)

#### Post-treatment

This stage is characterized by regular follow-ups and health assessments to ensure that cancer has not returned. Patients also receive medications or therapies to reduce the risk of recurrence, as well as breast reconstruction, if desired.

*Treatment fatigue and decision to discontinue*. The journey through cancer treatment is fraught with physical and emotional challenges that accumulate over time. For some Black participants, there comes a turning point where the prospect of additional treatment surpasses their threshold of what they can endure. In search of better options or second opinions, the patients may also seek out new doctors, which can lead to further delays in treatment and additional time lost in navigating the healthcare system.

“*I was unhappy with the oncologist because [he/she] didn’t have time to listen to me. I decided to find the second doctor*.” (Participant 1; Black participant)

“*The meeting was inappropriate and rude*. *I was exhausted thinking about it…So we thanked him for his time and we left there*. *I stayed awake all night thinking about it*. *The following week*, *I called the navigator to connect with another surgeon*” (Participant 8; Black participant)

“*It was enough to be dealing with what I was already dealing with*. *I wanted to give myself some time and not jump in right away*.” (Participant 10; Black participant)

*Patient autonomy*. Other participants find that they have an enhanced sense of control and empowerment over time in the later process. They emerge more informed and confident in making treatment-related decisions.

“*As we progress, I did my own research and took the initiative to share my findings with the professionals when deciding treatment choices…I felt more empowered.”* (Participant 4; Black participant)

“*I could have potentially had the reconstruction surgery*, *but I had my ovaries removed*. *I decided to just hold on*, *wait and do it next year*. *Because of my experience*, *I felt more empowered to make those kinds of decisions*.” (Participant 7; White participant)

*Follow-up care and continued support*. This theme explores the experiences of patients with the continuum of care following active treatment. Many participants emphasized the need for ongoing support and communication to address the needs of patients as they transition out of active treatment and into survivorship.

“*A follow-up call from the office after surgery would be helpful. I hoped to get some questions answered, but there was no ‘open-door’ post-visit.*” (Participant 1; Black participant)

“*After treatment*, *they teach you a little bit of stretches*. *But even this*. *They do this only when you demand it…That should be included in the treatment plan*, *like a follow up visit for physical therapy even if it’s just basic stretches*.” (Participant 7; White participant)

“*The nurse navigator was helpful*, *but once the chemotherapy is done*, *then you’re on your own*. *But I’m not done*. *I still need to start the oral chemotherapy drug*.” (Participant 7; White participant)

## Discussion

In this study, we have illuminated the complex journey of breast cancer patients in Indianapolis, with a focus on the differing experiences of Black and White women. Our findings reveal persistent disparities in the treatment process, echoing the national trend of higher mortality rates among Black women, despite lower incidence rates.

As reported in the literature, the breast cancer care continuum is a process in which feelings of anxiety [21], financial strain [22], loss of health and self-identity [23], physical changes [24], and changes in employment status may arise [25]. To provide cancer care that addresses the full spectrum of a patient’s needs, a deep understanding of their priorities and perceptions regarding treatment is essential. Mapping the patient’s journey proves invaluable in this context, facilitating a thorough exploration of the emotional, cognitive, and communal aspects of patient interactions within the multifaceted healthcare system [26]. Additionally, process analysis plays a key role in identifying and addressing delays, challenges, and bottlenecks at each stage, leading to optimizing each stage of treatment, and fostering multidisciplinary collaboration, and ultimately enhancing the quality and effectiveness of the care continuum.

This study underscores the impact of emotional responses and emphasizes the critical need for a robust support network throughout the cancer care continuum. Both Black and White participants consistently reported a lack of emotional support from healthcare professionals, reflecting a common theme in their experiences. These findings are supported by the literature, which highlights the importance of emotional support in cancer care. Slevin et al. noted that patients dissatisfied with their emotional support were significantly more likely to experience anxiety and depression [27]. Similarly, Wortman found that patients who received strong and consistent emotional support tended to adjust more successfully over time [28]. This reinforces the need for integrated emotional care as a fundamental part of the overall treatment strategy. Cancer care, which poses both emotional and physical challenges to patients and their families, requires that strategies be developed to deliver such care starting from primary care settings. A multidisciplinary professional team spanning various health service sectors is needed to meet these needs effectively.

Another theme consistently echoed in the experiences of both Black and White participants was the lack of effective patient-provider communication, with participants emphasizing the need to establish open lines of communication with their healthcare providers. Effective patient-provider communication is an essential component of patient care, facilitating a complete, accurate, timely, and unambiguous communication between providers and patient [29]. Its absence has been identified as a significant factor contributing to adverse outcomes. For example, Barlett et al. found that patients facing communication problems were at the highest risk for preventable adverse events due to poor clinical management and drug errors [30]. Patients have the right to be informed about their care, make educated decisions, and be listened to by their providers, but patient communication needs are often unmet or are inadequately addressed [29]. Reflecting on this, in a 2007 public policy paper focusing on health literacy, the Joint Commission recommended that healthcare organizations should make effective communication an organizational priority to protect patient safety and incorporate strategies to address patients’ communication needs across the continuum of care [31]. Recognizing effective patient-provider communication is a vital component, and it must be prioritized to improve patient safety.

The analysis of our study revealed distinct themes where the differences in experiences between Black and White participants were particularly pronounced. Closely related to ineffective patient-provider communication was delay in diagnosis. Despite the possible negative impact of delayed diagnosis and treatment on breast cancer survival, literature has shown that Black women continue to experience significantly longer delays in diagnosis and treatment compared to their White counterparts in current practice, even after adjusting for sociodemographic, clinical, and situational factors [32–34]. In alignment with the literature, our findings indicate a higher delay in the time intervals between different stages of the breast cancer continuum for Black participants compared to White participants. Furthermore, in addition to the systemic and insurance-related barriers faced by both Black and White participants, Black participants encountered an additional layer of barriers, influenced by a combination of provider and health system-related factors. In particular, many Black participants shared that they self-detected their cancer despite routine mammogram screenings and that their providers did not adequately explain the implications of their abnormal mammogram results, consequently delaying the treatment. Properly and effectively communicating abnormal mammograms is crucial–yet a gap often exists between what patients actually understand and what is conveyed by healthcare professionals, highlighting a critical area for improvement in patient education and provider training.

A theme that was particularly pronounced for Black participants is racial concordance. Our findings are consistent with existing research, suggesting that patient-provider communication is generally more satisfying in racially concordant dyads of minority populations, especially for Black patients [35, 36]. However, research spanning decades on concordance and physician-patient communication has produced conflicting results. Some studies, including a recent systematic review by Otte, have found minimal or no communication differences associated with concordance, and have deemed the relationship between patient outcomes and racial concordance inconclusive [37]. Conversely, other studies suggest that race and language concordance in patient navigators enhance the timeliness of care for minority populations [38]. These studies also note that patients who prefer racial concordance with their provider feel more at ease, find it easier to establish rapport, and appreciate the representation [39]. These findings underscore the need for a deeper understanding of provider support and social networks within the same race, advocating for the provision of individualized care where necessary.

We identified several barriers at systemic, social, and individual levels that influenced the experiences of Black women throughout the cancer care continuum. These factors were often interrelated and compounded in nature. Patient satisfaction decreased when they encountered barriers in any of these areas, and the challenges were exacerbated when multiple barriers were involved. Many Black participants sought a second opinion or decided to not receive further treatment as these challenges compounded. As such, although the reasons for seeking second opinions vary widely, studies suggest that patients’ primary motivations include a lack of trust, dissatisfaction with communication, a perceived need for certainty or confirmation, a need for more information, and a low understanding of the information provided [40–42]. It is also important to note that while obtaining second opinions provides patients with comprehensive treatment options, the process can delay treatment, as external imaging and records must be reviewed before presentation and assessment [43]. This underscores the importance of reducing adverse patient experiences through timely, effective communication and highlights the need for interventions to improve the referral process.

To our knowledge, this study is the first to compare the journey maps of Black and White breast cancer participants throughout the cancer care continuum. Our study illuminates the complex and varied experiences of breast cancer patients in Indianapolis, highlighting disparities between Black and White women. While both Black and White participants faced challenges related to financial barriers, treatment changes, lack of patient autonomy, and insufficient emotional support, Black participants additionally contended with barriers in cultural concordance and delays in diagnosis. These disparities mirror the national trend of higher mortality rates among Black breast cancer women. Central to these issues is the necessity for effective patient-provider communication and patient-centered care, which have proven to be crucial in influencing patient satisfaction, treatment timelines, and the overall quality of care. By addressing these challenges with equitable access to care and specific interventions, we can create a more equitable, responsive, and patient centered cancer care continuum, ultimately enhancing outcomes for all patients.

This study has certain limitations. The sample exclusively represents breast cancer survivors in the Indianapolis area, thus not fully capturing the breadth of socioeconomic disparities, as well as those faced in rural and other metropolitan areas. A larger national sample is required to comprehensively capture the disparities in care between Black and White breast cancer participants. Furthermore, qualitative research aims to understand specific phenomena and is not designed for generalization. Future research should focus on characterizing the barriers that lead to disparities in the care process throughout the patient journey, employing both quantitative and qualitative methods.

## Supporting information

S1 File Semi-structured interview guide for breast cancer participants. (DOCX)
