# Supplementary material for: Navigating the cancer care continuum: A comparative study of Black and White breast cancer patients
Source: PLoS One. 2024 Oct 24;19(10):e0312547. doi: 10.1371/journal.pone.0312547 (PMC11501014; doi:10.1371/journal.pone.0312547)
Supplement: S1 File — (DOCX) [file pone.0312547.s001.docx]

**S1 File. Semi-Structured Interview Guide for Breast Cancer Participants.**

*This guide outlines the primary topics for discussion with participants and these will be adjusted according to the patient’s individual language and situation.*

**Discovery and Initial Actions**

1. How did you discover your breast problem?
2. How long was it between the time you discovered your breast problem and the first time you went to a health provider about it?
3. How did you find a health provider to go to check out the problem?
4. What did the provider do after your exam?

**Screening and Diagnosis**

1. How was your appointment scheduled for your screening?
2. After your screening, how did you get your results?
3. How long was it between your screening and receiving the results of your screening?
4. Was the visit a positive or negative experience?
5. Did you get your questions answered during the visit?
6. Is physician/clinician visits a regular thing for you?
   What did you think of what medical professionals told you?
7. Did anyone attend this appointment with you?
8. How did their insurance coverage or other resources affect their decision making?

**Follow up and Treatment Planning**

1. How did you decide where and when to get follow-up tests for your breast problem?
2. Who, if anyone, explained to you what would happen at your follow-up tests?
3. How long was it between the date of your initial exam and the date of your follow-up tests?
4. Tell me as much as you can remember about what happened at your follow-up tests.
5. How long was it between the date of your follow-up tests and the date you received the results of your follow up tests?
6. How did you find out about your cancer diagnosis?
7. After learning of your diagnosis, who did you have conversation with to determine your next steps?

**Treatment**

1. What health providers were involved in your treatment, and how were you connected to them?
2. How long was it between the date you were told of your diagnosis and your first appointment to discuss treatment?
3. Did you receive any input or control over the dates and locations of your appointment?
4. If you had any problems during your treatment, who did you go for help?
5. Are you currently receiving any treatments or taking any medication related to your breast cancer diagnosis? If yes, what health providers are involved in your care?

**Conclusion**

Thank you for your time and for sharing your experiences with us today. Your insights are valuable and will contribute significantly to improving the experiences of others who may face similar situations in the future.
